# Health Economic Evaluation of Proton Therapy for Lung Cancer: A Systematic Review

**DOI:** 10.3390/ijerph20064727

**Published:** 2023-03-07

**Authors:** Chia-Chin Li, Ying-Chun Lin, Ji-An Liang, K. S. Clifford Chao, Te-Chun Hsia, Chun-Ru Chien

**Affiliations:** 1 Department of Radiation Oncology, China Medical University Hospital, Taichung 40402, Taiwan; 2 School of Medicine, College of Medicine, China Medical University, Taichung 40402, Taiwan; 3 Division of Pulmonary and Critical Care Medicine, Department of Internal Medicine, China Medical University Hospital, Taichung 40402, Taiwan; 4 Ph.D. Program for Health Science and Industry, College of Health Care, China Medical University, Taichung 40402, Taiwan

**Keywords:** health economic evaluation, lung cancer, proton therapy

## Abstract

Background: To our knowledge, there have been no systematic reviews of health economic evaluations of proton therapy specific to lung cancer. Methods: We conducted this systematic review according to the predefined protocol [PROSPERO CRD42022365869]. We summarized the results of the included studies via structured narrative synthesis. Results: We identified four studies (all used passively scattered proton therapy) from 787 searches. Two cost analyses reported that proton therapy was more costly than photon therapy for early- or locally advanced-stage non-small cell lung cancer, one cost-utility analysis reported that proton therapy was dominated by nonproton therapy in early-stage non-small cell lung cancer, and one cost-utility analysis reported that proton therapy was not cost-effective (vs. photon) in locally advanced non-small cell lung cancer. Conclusions: Passively scattered proton therapy was more costly and not cost-effective than photon therapy for early- and locally advanced-stage non-small cell lung cancer. Further health economic evaluations regarding modern proton therapy (such as scanning beam) for common radiotherapy indications of lung cancer are eagerly awaited.

## 1. Introduction

### 1.1. Lung Cancer Radiotherapy

Lung cancer is one of the leading causes of cancer mortality worldwide [1]. Lung cancer is usually classified as non-small cell lung cancer (NSCLC, the majority) or small cell lung cancer (SCLC). Radiotherapy is a common modality in the treatment of lung cancer, whether for SCLC or NSCLC [2,3,4]. Currently, radiotherapy is usually delivered via photons [5,6]. There are many photon radiotherapy technologies available in the treatment of lung cancer, including the minimal standard of 3-dimensional conformal radiotherapy (3D-CRT, also called 3DRT) [2,3,4,7] or more advanced technologies such as intensity-modulated radiotherapy (IMRT).

### 1.2. Proton Therapy Is an Advanced Radiotherapy Technology

**Figure 1:**
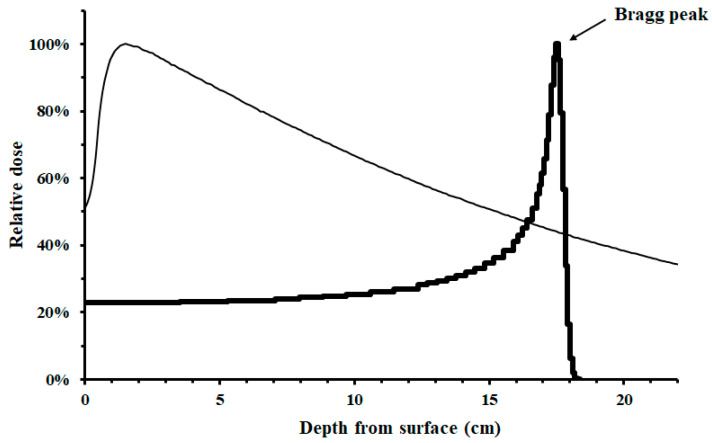
Illustrative dose distribution of proton therapy (bold line) vs. photon therapy (thin line).

With the theoretical benefit of a lower normal tissue dose, proton therapy is advocated as an advanced form of radiotherapy [6] and may be used to limit normal tissue toxicity to deliver radiotherapy safely [3,4,8]. This benefit was contributed by the physical characteristics (Bragg peak) of particle therapy (including protons or carbon ions), which are not available in commonly used photon radiotherapy [6,9]. As shown in Figure 1, proton therapy typically delivered most of the radiotherapy dose at the desired body depth (Bragg peak) with much lower dose before and almost no dose after that depth. On the contrary, photon therapy delivered much of the dose close to body surface (usually not the desired depth). Therefore, the use of proton therapy has been expanded from the 1st hospital based one in 1990 to more than 100 proton centers in operation around the world [6] and advocated for the treatment of various cancer types, including lung cancer [3,4,10]. However, the technology for delivering protons is evolving as well, similar to the history of delivering photon radiotherapy, which has evolved from 3DRT to IMRT. In the past, most proton therapy was delivered via passively scattered proton therapy (PSPT), whereas the use of scanning beam proton therapy has emerged to deliver intensity-modulated proton therapy (IMPT) [6]. In the field of thoracic radiation oncology, an earlier randomized controlled trial (RCT) using PSPT vs. IMRT reported similar results for locally advanced NSCLC (LA-NSCLC) [11], whereas a more recent RCT allowing scanning beam proton therapy [12] reported significantly fewer side effects while maintaining similar progression-free survival (PFS) when proton therapy was compared with IMRT in the treatment of locally advanced esophageal cancer. New RCTs (such as NCT01993810) allowing scanning beam proton therapy in the treatment of lung cancer are ongoing [6].

### 1.3. Health Economic Evaluation of Proton Therapy for Lung Cancer

Financial toxicity has been a common challenge to modern cancer therapy [13,14]. Due to the high cost of proton therapy with initial investment far greater than that of a photon unit [6,15], health economic evaluation (HEE) is of tremendous importance in the evaluation of proton therapy [15,16]. An earlier systematic review found no papers reporting the cost-effectiveness of particle therapy [17]. Since then, to our knowledge, no systematic review specifically of HEE of lung cancer has been published, although HEE or the cost-effectiveness of proton therapy in general (not limited to lung cancer) has been reviewed [15,16,17,18,19]. However, the most recent systematic review was conducted in July 2019 [16], more than three years ago. Furthermore, this systematic review only focused on the narrow cost-effectiveness analyses rather than the broader HEE [20,21,22]. Therefore, the aim of our study was to systematically review the health economic evaluation of lung cancer patients treated with proton vs. alternative radiotherapeutic approaches from which future research priorities can be identified.

## 2. Materials and Methods

We conducted this systematic review according to the predefined protocol [PROSPERO CRD42022365869, registered in Oct 2022]. The target population was adult lung cancer patients treated with radiotherapy via either proton (monotherapy or combination therapy with other treatment modalities) or nonproton (including but not limited to photons or carbon ions). The inclusion criteria were health economic evaluation [20,21,22] in original full English papers that reported the economic (with/without health) outcomes of proton and nonproton therapies without limitations regarding time horizon, study perspective or study design. We searched PubMed, EMBASE, and Cochrane during October 2022 for the initial search and January 2023 for the final search. The selection of these databases was based on recommendations in the literature [23] and our previous experience [24]. We used the following search strategy in PubMed as modified from the literature [25] and our previous experience [24]: (lung) AND ((carcinoma) OR (neoplasm) OR (cancer)) AND (proton) AND ((“costs and cost analysis” [MeSH] OR costs [Title/Abstract] OR cost effective* [Title/Abstract]) OR (cost* [Title/Abstract] OR “costs and cost analysis” [MeSH:noexp] OR cost benefit analysis* [Title/Abstract] OR cost–benefit analysis [MeSH] OR health care costs [MeSH:noexp])). We modified the strategy for EMBASE as follows: (‘lung’/exp OR lung) AND (‘carcinoma’/exp OR carcinoma OR ‘neoplasm’/exp OR neoplasm OR ‘cancer’/exp OR cancer) AND (‘proton’/exp OR proton) AND (‘cost’/exp OR cost). We modified the strategy for Cochrane as follows: “((lung) AND ((carcinoma) OR (neoplasm) OR (cancer)) AND (proton) AND (cost))”. We performed further supplementary searching by checking the bibliographies of all included studies and systematic reviews identified via the above search in PubMed. We followed the PRISMA 2020 flow diagram [26,27] to identify studies to be included in our systematic review. After the above study identification from the search, duplicated records were excluded, and two reviewers independently screened the title and abstract to identify potentially eligible reports for full paper retrieval. Of the retrieved reports, we screened full-text articles to determine if they met the criteria to be included in the final analyses. We used CHEERS 2022 [20] for quality assessment of the included studies and then synthesized the results of the included studies via structured narrative synthesis (tabulation, see Section 3 for details). For the convenience of the intended readers around the world, costs (rounded as integral) were normalized to 2023 USD by the inflation factor and Purchasing Power Parities using the CCEMG–EPPI-Centre Cost Converter (https://eppi.ioe.ac.uk/costconversion/, accessed on 10 January 2023) in the summary tables, whereas both the updated dollar values and the original (shown in square brackets if different from the updated value) were reported in the text. We extracted the following elements into the summary table, as modified from the literature and our experiences [23,24]: author, study year, type of health economic evaluation, conflict of interest, source of funding, studied population, ethnicity, country, treatment setting, study design, analytic approach, study perspective, time horizon, discount rate, type of costs, cost year, type of effectiveness, compliance with treatment, statistical software, intervention and comparator, (incremental) cost, incremental cost-effectiveness ratio, (incremental) effectiveness, sensitivity analyses, and conclusion. We used the year reported in the paper as the reference year for cost. If such information was not reported, we then used the reference year based on the most relevant year of data used or the year in which the paper was published [24]. All the above assessments and data extraction were performed by two reviewers (Li C.C. & Chien C.R.) independently, with input from the third (Lin Y.C.) if there was no consensus after face-to-face discussion between Li and Chien.

## 3. Results

**Figure 2:**
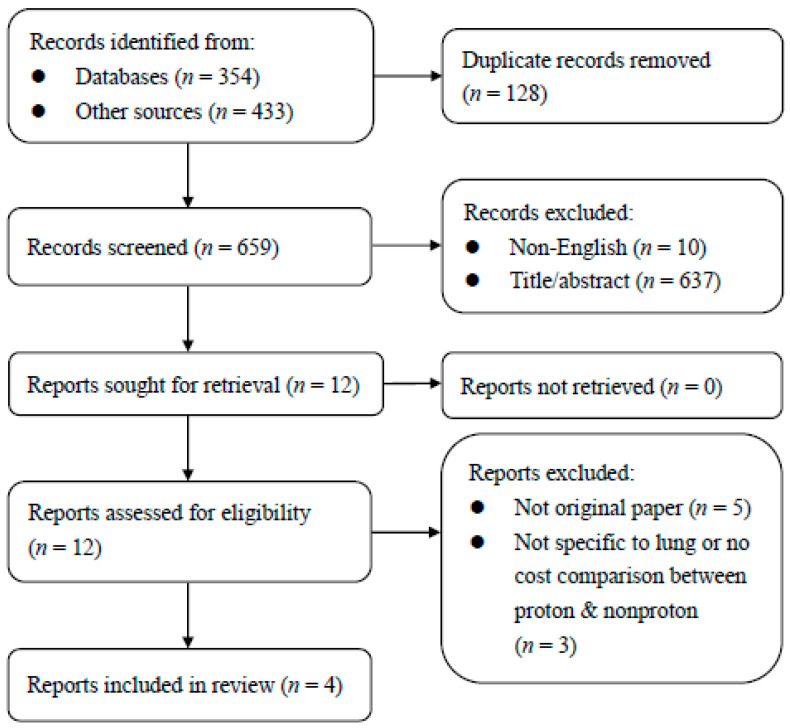
Flow diagram of the literature search.

After the final search in January 2023, the flow diagram of the literature search (combining the initial and final searches) is illustrated in Figure 2. Among the 787 searches, we included four studies in our final analyses [28,29,30,31].

We determined that there was high variability in clinical (stage I vs. III) and health settings (Netherland vs. USA with time span from 2007~2019) among these included studies, so we performed the planned structured narrative synthesis of available studies as a summary table (Table 1) rather than formal meta-analyses [23]. There were two cost analyses and two cost-utility analyses. Two study [28,31] investigated definitive radiotherapy +/− chemotherapy for LA-NSCLC (stage III) in the US or the Netherlands, whereas two studies [29,30] investigated definitive radiotherapy for early-stage (stage I) NSCLC in the Netherlands. All studies investigated passively scattered proton therapy, employed modeling approaches, and found that PSPT was more costly than photon therapy (Table 1). Two studies [29,30] (from the same study group) stated that “cost difference between particle and photon therapies is relatively small for lung cancer” but “proton therapy was dominated by both carbon-ion therapy and stereotactic body radiotherapy (SBRT)” for early-stage NSCLC. One study [28] reported that protons were more costly than photons (48,559 vs. 22,767 vs. 30,593 (42,975 vs. 20,149 vs. 27,075 USD) for proton vs. photon-3DCRT vs. photon-IMRT) in definitive concurrent chemoradiotherapy (CCRT) for LA-NSCLC. Even in the group with the highest risk of radiation pneumonitis in which proton therapy might lead to additional cost savings, proton therapy was still much more costly than either IMRT or 3DCRT. In another study investigating radiotherapy for LA-NSCLC [31], the authors stated “Currently, proton therapy is not cost-effective for all patients”. However, “individualized proton” may be cost-effective at threshold of 104,121 USD [EUR 76,299] per QALY if equal minutes per fraction for either proton or photon.

The quality assessment is shown in Supplementary Table S1. All studies reported (at least partially) most of the required items suggested in the literature [20]. Heterogeneity was only explored in one study [28] in which the results remained similar.

## 4. Discussion

We systematically reviewed the published HEEs regarding proton therapy for lung cancer. In addition to the CEA published in 2010 [30], which was summarized by the most recent systematic review in general (not specific for lung cancer) [16], we further summarized other broader [28,29] or more recent [31] HEEs. We found that the available HEEs regarding proton therapy for lung cancer were limited to specific scenarios of lung cancer radiotherapy, whereas no HEE was available for the vast majority of common radiotherapy indications for lung cancer (Table 2) [2,32]. Our study was, to our knowledge, the first systematic review specifically for HEE of lung cancer proton therapy.

The results summarized in our systematic review seem to be relatively homogenous (regardless of whether sponsorship was reported or not [23]) in that proton therapy was more costly than photon therapy, as expected. However, there were only four studies included, and the investigated scenarios were limited (Table 2), so this observation should be interpreted with caution with limitations in generalizability. Furthermore, the clinical and health setting was quite variable [23] and limited (Table 2).

There were some limitations to our study. First, we did not use gray literature as emphasized in the literature [23] because it was potentially nonreproducible [23] and, thus, less scientific to our way of thinking. Second, we did not consider the risk of bias in the included studies during data synthesis. This was because the risk of bias was not mentioned in the modern CHEERS 2022 checklist [20] for HEE. Furthermore, there were only four studies included in our study, and the clinical and health settings were quite variable, as mentioned above. Finally, we did not identify HEEs investigating more modern proton therapy such as scanning beam protons [6] or magnetic resonance guided proton therapy [33,34]. When more clinical results of modern proton therapy are available in the future, the cost and cost effectiveness of modern proton therapy may be clarified in the future. These relied on the collaborative work among various stakeholders, including patients, health care professionals, manufactures, insurance agencies, and the governments. The future of proton therapy for lung cancer also relates to the advancement of other treatment modalities such as radiofrequency ablation [35], surgery [36], and systemic therapy [37,38].

## 5. Conclusions

In this systematic review, we found that PSPT was reported to be dominated by both carbon-ion therapy and photons (SBRT) for early-stage NSCLC and was also more costly and not as cost effective than photons for locally advanced-stage NSCLC. There were no HEEs available for the vast majority of common radiotherapy indications for lung cancer. Further HEEs regarding modern proton therapy (such as scanning beam) for common radiotherapy indications of lung cancer are eagerly awaited.

## Figures and Tables

**Table 1:** Summary table of the included health economic evaluations (HEE).

Study	Population	Analyses	I & C	Main Outcomes	Other Results & SA	Conclusion & Note
Peeters et al., 2010 [29] # HEE: cost analysis # COI: NR # SOF: NR	# CSP: inoperable stage I NSCLC, Netherland # age: NR # gener: NR # ethnicity: NR # TS: definitive RT	# SD-AA: modeling (linear) # SP: health-care perspective (hospital) # TH: during treatment # DR: NA # TC: direct medical (capital and operational) # Cost year: 2007 EUR # TE: NA # CWT: NA # SW: Excel	Proton ^※^ (only, DOT 2) vs. Proton ^※^ (combined with carbon-ion, DOT 2) vs. NP (carbon-ion, DOT 1) vs. NP (photon-3DRT, DOT 7) vs. NP (photon-SBRT, DOT 1)	# CO: 19,195 vs. 29,148 vs. 15,551 vs. 12,636 vs. 5768	# cost range: (19,195–38,389) vs. (29,148–58,297) vs. (3892–69,956) vs. (1442–14,435) vs. (7225–15,877) # DSA: NR # PSA: NR	# “Cost difference between particle and photon therapies is relatively small for lung cancer” # Note: cost was calculated by the number of fractions and time to deliver a single fraction with an expected minimal treatment cost of EUR 2500; also reported cost for RT indications for other cancers.
Grutters et al., 2010 [30] # HEE: CUA # COI: no # SOF: Siemens Medical Solutions.	# CSP: inoperable stage I NSCLC, Netherland # age: NR # gener: NR # ethnicity: NR # TS: definitive RT	# SD-AA: modeling (decision-analytic Markov model) # SP: health-care perspective # TH: 5 years # DR: 1.5% (effect) & 4% (cost) # TC: direct medical cost of RT plus cost for AE and FU # cost year: 2007 EUR # TE: QALY # CWT: NA # SW: Excel	Proton ^※^ (DOT 3) vs. (NP: carbon-ion, DOT 1) vs. (NP: CFRT, DOT 7) vs. (NP: SBRT, DOT 1)	# CO: 42,741 vs. 29,792 vs. 35,189 vs. 21,519 # HO: 2.33 vs. 2.67 vs. 1.98 vs. 2.59 # ICER: $21,578 per QALY (proton vs. CFRT)	# Proton therapy was dominated by both carbon-ion therapy and SBRT. # DSA: NR # PSA: 2% probability of being cost-effective at WTP EUR 80,000 [USD124,036] per QALY # Additional SA: ICER of USD126,329 per QALY gained for proton vs. carbon-ion if alternative parameters.	# “it is recommended not to adopt particle therapy as standard treatment” # EVPI of adopting particle therapy: “EUR 22 million” [USD34 million] # Note: Some of the assumptions (such as no recovery from ≥grade 3 dyspnea) may not be logical.
Smith et al., 2018 [28] # HEE: cost analysis # COI: no # SOF: NR	# CSP: stage III NSCLC, USA # age: NR # gener: NR # ethnicity: NR # TS: definitive CCRT	# SD-AA: modeling (influence diagram) # SP: health-care perspective (third-party payer, Medicare) # TH: 6 months # DR: NA # TC: direct medical cost (RT and AE) # Cost year: 2017 USD # TE: NA # CWT: NA # SW: NR	Proton (passively scattered, DOT 6) vs. NP (photon-3DRT, DOT 6) vs. NP (photon-IMRT, DOT 6)	# CO: (combined RT & AE) 48,559 vs. 22,767 vs. 30,593	# (for high-risk group): IC: 25,530 (proton vs. 3DRT) or 17,856 (proton vs. IMRT) # DSA: NR # PSA: NR	# “Current costs favor X-ray therapy. However, relatively small reductions in the cost of proton therapy may result in a shift to the preference for proton therapy” # Note: also reported analyses if risk-adjusted or biomarker available.
Aldenhoven et al., 2022 [31] # HEE: CUA # COI: NR # SOF: nil	# CSP: NSCLC stage III, Netherland # age: mean 66 years old # gener: male 69.2% # ethnicity: NR # TS: radiotherapy +/− chemotehrapy	# SD-AA: modelling (decision-analytical state-transition) # SP: Societal # TH: life time # DR: 1.5% (effect) or 4% (cost) # TC: direct medical, direct nonmedical, and indirect (see its Table 4) # Cost year: 2019 EUR # TE: QALY # CWT: NA # SW: Excel	“all proton” (passively scattered, DOT 6) vs. photon ( DOT 6) vs. “individualized proton” (passively scattered, DOT 6) (by estimated toxicity risk)	# CO: 108,755 vs. 56,265 vs. 94,029 # HO: 1.951 vs. 1.769 vs. 1.922	# DSA: cardiac event categories and “no toxicity” utilities were the most influential parameters # PSA: photon had the highest probability of being cost-effective (97%) at a threshold of EUR 80,000 [USD109,171] per QALY # (vs. photon) “individualized proton” or “all proton” was cost-effective at threshold of EUR 163,467 [USD223,074] or 301,396 [USD411,297] per QALY, respectively # (vs. photon) “individualized proton” will be cost-effective at threshold of EUR 76,299 [USD104,121] per QALY if equal minutes per fraction for either proton or photon	# “Currently, PT is not cost-effective for all patients” # Note: @ issue-1: The current standard of care [consolidative immunotherapy for those progression-free after CCRT] was not considered in the model. @ issue-2: neglected the landmark randomized controlled trial [NCT00915005]. @ issue-3: The accuracy of its Table 1 regarding the efficacy of proton was concerned because the improvement in normal organ dose in the cited reference [ROCOCO] was not that obvious in the above real trial and the improvement in survival modelling was not observed in the above real trial.

3DRT: three-dimensional RT; AE: adverse events; CCRT: concurrent chemoradiotherapy; CFRT: conventional fractionated radiotherapy via photon; CO: cost outcomes; COI: conflicts of interest; CSP: country of studied population; CUA: cost-utility analyses; CWT: compliance with treatment; DOT: duration of treatment (weeks in integer, assuming five fractions per week); DR: discount rate; DSA: deterministic SA; EVPI: expected value of perfect information; FU: follow-up; HO: health outcomes; I & C: intervention and comparator; IC: incremental cost; ICER: incremental cost-effectiveness ratio; IMRT: intensity modulated radiotherapy; NA: not applicable; NP: nonproton; NR: not reported; NSCLC: non-small cell lung cancer; PSA: probabilistic SA; QALY: quality adjusted life years; RT: radiotherapy; SA: sensitivity analyses; SBRT: stereotactic body radiotherapy via photon; SD-AA: study design & analytic approach: SOF: sources of funding; SP: study perspective; SW: statistical software; TC: type of costs [direct costs, capital costs; Indirect medical costs; indirect costs]; TE: type of effectiveness; TH: time horizon; TS: treatment setting; WTP: willingness to pay; ^※^ assuming passively scattered because the referenced proton data from publications; # subsection under each column.

**Table 2:** Summary table by common lung cancer radiotherapy indications [modified from [2,3,4,31]].

Disease	Indication & Treatment Setting	Comparison	HEE	Note
NSCLC	Inoperable early stage (mainly stage I): definitive RT [29,30]	Proton ^※^ vs. photon or carbon ion	Proton therapy was dominated by both carbon-ion therapy and photon (SBRT).	Proton therapy may be cost-effective when compared to CFRT
NSCLC	Inoperable LA stage (mainly stage III): definitive CCRT [28,31]	Proton ^※^ vs. photon	Proton was more costly and not cost-effect when compared to photon	“individualized proton” may be cost-effective if equal minutes per fraction for either proton or photon
NSCLC	Oligometastatic stage IV: curative radiotherapy (with or without SBRT)	^#^	^#^	^#^
NSCLC	Polymetastatic stage IV: palliative radiotherapy (such as for LMM)	Proton vs. photon	^#^	^#^; Scanning beam proton therapy is more effective than photon for NSCLC or breast cancer patients with LMM [NCT04343573]
NSCLC	Locoregional recurrence without prior irradiation	^#^	^#^	^#^
NSCLC	Locoregional recurrence after prior irradiation	^#^	^#^	^#^
SCLC	LS: definitive CCRT	^#^	^#^	^#^
SCLC	ES: consolidative thoracic radiotherapy	^#^	^#^	^#^
SCLC	ES: palliative radiotherapy (such as for thorax)	^#^	^#^	^#^
SCLC	LS or ES: PCI	^#^	^#^	^#^

^#^ no relevant study found in the current systematic review so there were no references in this row; ^※^ passively scattered; 3DRT: three-dimensional RT; CCRT: concurrent chemoradiotherapy; CFRT: conventional fractionated radiotherapy via photon; ES: extensive stage; HEE: health economic evaluation; IMRT: intensity-modulated radiotherapy; LA: locally advanced; LMM: leptomeningeal metastasis; LS: limited stage; NSCLC: non-SCLC; PCI: prophylactic cranial irradiation; RT: radiotherapy; SBRT: stereotactic body radiotherapy (also called stereotactic ablative radiotherapy, SABR); SCLC: small cell lung cancer.

## Data Availability

The data presented in this study are available on request from the corresponding author.

## Supplementary Materials

The following supporting information can be downloaded at: https://www.mdpi.com/article/10.3390/ijerph20064727/s1, Table S1. Quality assessment of the included studies.
