# Supplementary material for: Health Economic Evaluation of Proton Therapy for Lung Cancer: A Systematic Review
Source: Int J Environ Res Public Health. 2023 Mar 7;20(6):4727. doi: 10.3390/ijerph20064727 (PMC10048835; doi:10.3390/ijerph20064727)
Supplement: Supplementary file 1 [file ijerph-20-04727-s001.zip › ijerph-2194255-supplementary.pdf]

Table S1. Quality assessment of the included studies.

Section/topic	Item No	Guidance for reporting	Reported in section			
			Smith et al. 2018 [28]	Peeters et al. 2010 [29]	Grutters et al. 2010 [30]	Aldenhoven et al. 2022 [31]
Title						
Title	1	Identify the study as an economic evaluation and specify the interventions being compared.	Title	Title	Title	Title
Abstract						
Abstract	2	Provide a structured summary that highlights context, key methods, results, and alternative analyses.	Abstract	Abstract	Abstract	Abstract
Introduction						
Background and objectives	3	Give the context for the study, the study question, and its practical relevance for decision-making in policy or practice.	Introduction	Introduction	Introduction	Introduction
Methods						
Health economic analysis plan	4	Indicate whether a health economic analysis plan was developed and where available.	Not reported	Not reported	Not reported	Not reported
Study population	5	Describe characteristics of the study population (such as age range, demographics, socioeconomic, or clinical characteristics).	Methods: Model design	Analyses: Treatment scenarios	Methods	Methods
Setting and location	6	Provide relevant contextual information that may influence findings.	Methods	Methods	Methods	Methods
Comparators	7	Describe the interventions or strategies being compared and why chosen.	Methods: Model design	Analyses: Treatment scenarios	Methods: Model description	Methods: State-transition model approach and structure
Perspective	8	State the perspective(s) adopted by the study and why chosen.	Methods: Model assumptions	Methods: Sources of estimates	Methods: Costs	Methods: State-transition model approach and structure
Time horizon	9	State the time horizon for the study and why appropriate.	Methods: Model design;	Analyses	Methods: Model description	Methods: State-transition model approach and structure
Discount rate	10	Report the discount rate(s) and reason chosen.	NA	NA	Methods: Effects & Costs	Methods: State-transition model approach and structure

Selection of outcomes	11	Describe what outcomes were used as the measure(s) of benefit(s) and harm(s).	Methods: Types of analyses	Analyses: Treatment scenarios	Methods: Effects & Costs	Methods
Measurement of outcomes	12	Describe how outcomes used to capture benefit(s) and harm(s) were measured.	Methods: Types of analyses	Analyses	Methods: Effects & Costs	Methods
Valuation of outcomes	13	Describe the population and methods used to measure and value outcomes	Methods: Types of analyses	Analyses	Methods: Analysis	Methods
Measurement and valuation of resources and costs	14	Describe how costs were valued.	Methods: Determination of costs	Analyses	Methods: Costs	Methods: Health state and event resource use and costs
Currency, price date, and conversion	15	Report the dates of the estimated resource quantities and unit costs, plus the currency and year of conversion.	Methods: Determination of costs	Methods	Methods	Methods: Health state and event resource use and costs
Rationale and description of model	16	If modeling is used, describe in detail and why used. Report if the model is publicly available and where it can be accessed.	Methods: Model design	Analyses: Treatment scenarios	Methods: Model description	Methods: State-transition model approach and structure
Analytics and assumptions	17	Describe any methods for analyzing or statistically transforming data, any extrapolation methods, and approaches for validating any model used.	Methods	Methods & Analyses	Methods: Analysis	Methods
Characterizing heterogeneity	18	Describe any methods used for estimating how the results of the study vary for subgroups.	Methods: Types of analyses	Not reported	Not reported	Not reported
Characterizing distributional effects	19	Describe how impacts are distributed across different individuals or adjustments made to reflect priority populations.	Not reported	Not reported	Not reported	Not reported
Characterizing uncertainty	20	Describe methods to characterise any sources of uncertainty in the analysis.	Not reported	Not reported	Methods: Analysis	Methods: Sensitivity analysis
Approach to engagement with patients and others affected by the study	21	Describe any approaches to engage patients or service recipients, the general public, communities, or stakeholders (such as clinicians or payers) in the design of the study.	Not reported	Not reported	Not reported	Not reported
Results						
Study parameters	22	Report all analytic inputs (such as values, ranges, references), including uncertainty or distributional assumptions.	Methods: determination of costs & Types of analyses; Results: Radiation and toxicity costs	Analyses: Treatment scenarios	Table 1	Methods: (table 1 – 4)
Summary of main results	23	Report the mean values for the main categories of costs and outcomes of interest and summarize them in the most appropriate overall measure.	Results: Rate-adjusted analysis	Results: Treatment scenarios	Results	Results

Effect of uncertainty	24	Describe how uncertainty about analytic judgments, inputs, or projections affect findings. Report the effect of choice of discount rate and time horizon, if applicable.	Not reported	Not reported	Results: Figure 2 & Sensitivity analysis	Results
Effect of engagement with patients and others affected by the study	25	Report on any difference patient/service recipient, general public, community, or stakeholder involvement made to the approach or findings of the study.	Not reported	Not reported	Not reported	Not reported
Discussion						
Study findings, limitations, generalizability, and current knowledge	26	Report key findings, limitations, ethical or equity considerations not captured, and how these could affect patients, policy, or practice	Discussion	Discussion	Discussion	Discussion
Other relevant information						
Source of funding	27	Describe how the study was funded and any role of the funder in the identification, design, conduct, and reporting of the analysis	Not reported	Not reported	Acknowledgment	Funding
Conflicts of interest	28	Report author conflicts of interest according to journal or International Committee of Medical Journal Editors requirements	Footnote	Not reported	Conflict of interest statement	Not reported

NA: Not applicable.
